# Synthesis, Characterization, Magnetic Properties, and Applications of Carbon Dots as Diamagnetic Chemical Exchange Saturation Transfer Magnetic Resonance Imaging Contrast Agents: A Review

**DOI:** 10.3390/nano15070542

**Published:** 2025-04-02

**Authors:** Endale Mulugeta, Tirusew Tegafaw, Ying Liu, Dejun Zhao, Ahrum Baek, Jihyun Kim, Yongmin Chang, Gang Ho Lee

**Affiliations:** 1 Department of Chemistry, College of Natural Sciences, Kyungpook National University, Taegu 41566, Republic of Korea; endexindex05@gmail.com (E.M.); tirukorea@gmail.com (T.T.); ly1124161@gmail.com (Y.L.); djzhao.chem@gmail.com (D.Z.); 2 Institute of Biomedical Engineering Research, Kyungpook National University, Taegu 41944, Republic of Korea; baxun@naver.com; 3 Department of Chemistry Education, Teachers’ College, Kyungpook National University, Taegu 41566, Republic of Korea; jkim23@knu.ac.kr; 4 Department of Molecular Medicine, School of Medicine, Kyungpook National University, Taegu 41944, Republic of Korea

**Keywords:** carbon dot, synthesis, characterization, magnetic properties, diaCEST, MRI contrast agent

## Abstract

Carbon dots (CDs) are metal-free carbon-based nanoparticles. They possess excellent photoluminescent properties, various physical properties, good chemical stability, high water solubility, high biocompatibility, and tunable surface functionalities, suitable for biomedical applications. Their properties are subject to synthetic conditions such as pH, reaction time, temperature, precursor, and solvent. Until now, a large number of articles on the synthesis and biomedical applications of CDs using their photoluminescent properties have been reported. However, their research on magnetic properties and especially, diamagnetic chemical exchange saturation transfer (diaCEST) in magnetic resonance imaging (MRI) is very poor. The diaCEST MRI contrast agents are based on exchangeable protons of materials with bulk water protons and thus, different from conventional MRI contrast agents, which are based on enhancements of proton spin relaxations of bulk water and tissue. In this review, various syntheses, characterizations, magnetic properties, and potential applications of CDs as diaCEST MRI contrast agents are reviewed. Finally, future perspectives of CDs as the next-generation diaCEST MRI contrast agents are discussed.

## 1. Introduction

**Figure 1:**
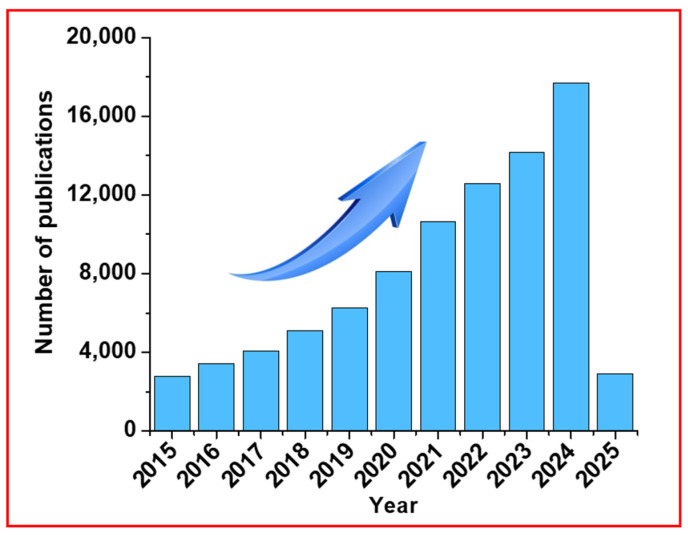
The annual number of research articles related to CDs (the statistical data are from the Scopus database up to February 2025).

Carbon dots (CDs) were noticed ~20 years ago and exhibit emission in the visible region [1]. Sun et al. prepared bright and colorful photoluminescent CDs by a laser ablation method from graphite powder and cement [2]. CDs have great potential for applications in biomedical and environmental areas [3,4,5,6]. For example, bioimaging and chemo-sensing, cellular imaging, drug delivery, cancer therapy, pollutant removal, wastewater treatment, and environmental remediation are the possible application areas [7,8,9,10,11,12,13]. Moreover, surface composition engineering through post-synthetic approaches allows for the expansion and optimization of the range of CD applications. Recently, CDs have received great attention owing to their simple synthesis and surface functionalization, good biocompatibility, and highly stable emission [14]. CDs can contain various surface functional groups such as hydroxyl (-OH), carboxyl (-COOH), carbonyl (-CO), and amine (-NH_2_) groups, which are suitable for further surface functionalizations [15,16,17]. Surface functional groups enhance the reactivity of CDs towards conjugation reactions and tune their surface properties. Surface functionalization of CDs with oxygen-containing groups tends to exhibit negatively charged surfaces, while nitrogen-containing functional groups can result in positively charged surfaces, making CDs stable colloids in aqueous media. Since the discovery of CDs, two decades have gone by and a lot of CD-based research papers have been published [18]. To highlight the importance of CD-based studies and progress, we used the Scopus database to find out the total number of articles related to CDs published for the last ten years (≥2015). Figure 1 displays the number of research papers related to CDs and definitely reveals that the trend keeps on increasing every year. Among them, publications on CDs as diaCEST MRI contrast agents are not rich; until now, there exist only a few studies [19,20,21,22]. Therefore, continuing studies on this subject are critically needed.

So far, CDs have been applied to various biomedical areas [7,8,9,10,11,23]. However, few application studies on contrast agents in magnetic resonance imaging (MRI) exist. MRI is one of the primary techniques used in disease detection, diagnosis, and monitoring. Over the last decades, MRI technology has been continuously improved [24]. These improvements include the enhancement of the image clarity, reduction in the scan times, and development of the high-field scanners. Furthermore, MRI image qualities and diagnostic precisions have been significantly improved with the development of contrast agents. Among them, a new class of metal-free MRI contrast agents based on chemical exchange saturation transfer (CEST) had been introduced by Ward et al. [25]. This CEST works through the proton exchange of contrast agents with bulk water protons to enhance image contrasts at the accumulated region of the contrast agents.

In general, the CEST MRI contrast agents can be divided into two groups based on their compositions [25,26,27]: paramagnetic CEST (paraCEST) MRI contrast agents, which are paramagnetic metal ion (Eu^3+^, Dy^3+^, etc.) complexes, and diamagnetic CEST (diaCEST) MRI contrast agents, which are made of metal-free materials with exchangeable protons with bulk water protons. In this review, various synthetic approaches and characterization techniques of CDs are briefly reviewed. Then, magnetic properties and applications of CDs as diaCEST MRI contrast agents as a new class of metal-free MRI contrast agents are discussed and highlighted along with their future perspectives.

## 2. Synthesis of CDs

**Figure 2:**
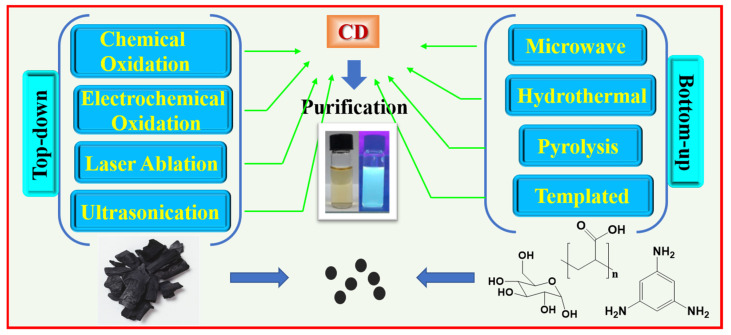
Schematic representation of “top-down” and “bottom-up” approaches in CD syntheses.

Until now, various synthetic approaches of CDs have been reported. A well-established synthesis method will afford CDs with uniform size, high quantum yield (QY), and scalable and cost-effective production. CDs can be prepared largely by two approaches: “top-down” and “bottom-up”, as depicted in Figure 2.

### 2.1. Top-Down Approach

**Figure 3:**
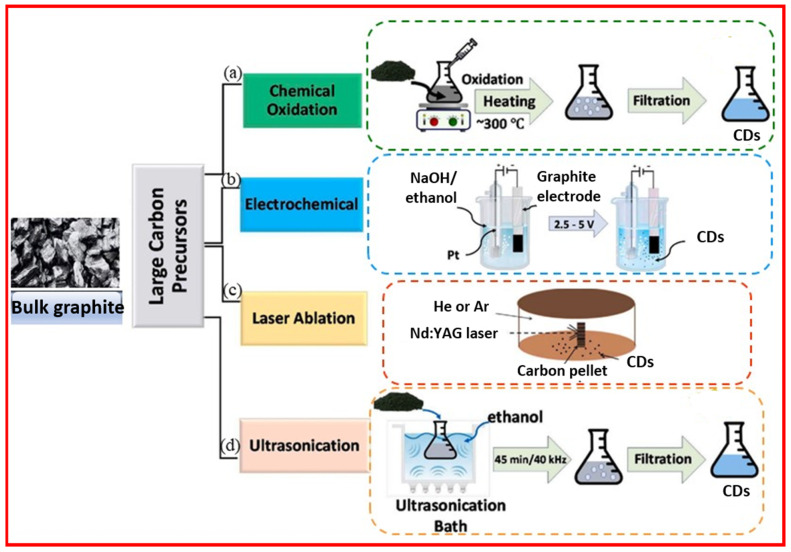
Schematic representation of top-down approaches of CD synthesis: (**a**) chemical oxidation, (**b**) electrochemical oxidation, (**c**) laser ablation, and (**d**) ultrasonication. Reproduced with permission [29].

The top-down approach involves cleavage and exfoliation of carbon precursors such as graphite powder, activated carbon, carbon black, carbon nanotubes, and carbon fibers [28]. The top-bottom approach includes chemical oxidation, electrochemical oxidation, laser ablation, and ultrasonication as depicted in Figure 3.

#### 2.1.1. Chemical Oxidation

The chemical oxidation method utilizes strong oxidants such as HNO_3_ and H_2_SO_4_ to oxidize carbon precursors to prepare CDs [30]. Chemical oxidation is a facile and convenient method for mass production of CDs.

**Figure 4:**
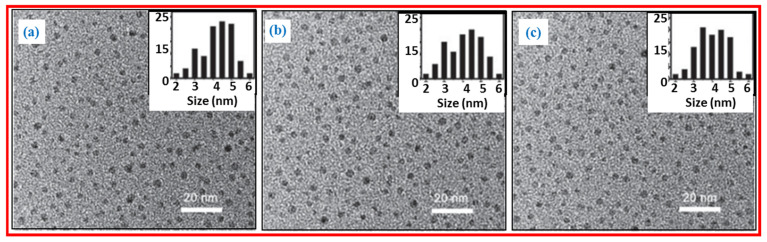
TEM images and size distributions of CDs prepared from (**a**) CAC, (**b**) WAC, and (**c**) CAC precursors. Reproduced with permission [31].

Qiao et al. prepared multicolor photoluminescent CDs from three different activated carbon precursors by chemical oxidation [31]. Coal-activated carbon (CAC), wood-activated carbon (WAC), and coconut-activated carbon (CAC) were treated with HNO_3_ to obtain CDs. Further, the CDs were coated with amine-terminated compounds. Figure 4a–c exhibits TEM images and size distribution of CDs prepared from CAC, WAC, and CAC precursors; they had average diameters of 4.5 ± 0.6 nm, 4.2 ± 0.8 nm, and 4.2 ± 0.6 nm, respectively. The CDs were water-soluble and displayed multicolor photoluminescent properties with high quantum yields and good biocompatibility.

#### 2.1.2. Electrochemical Oxidation

Electrochemical oxidation is suitable to tune the properties of CDs by controlling electrochemical parameters, solvent, and carbon precursors [32]. The size of CDs can be tuned by changing the applied potential [33].

**Figure 5:**
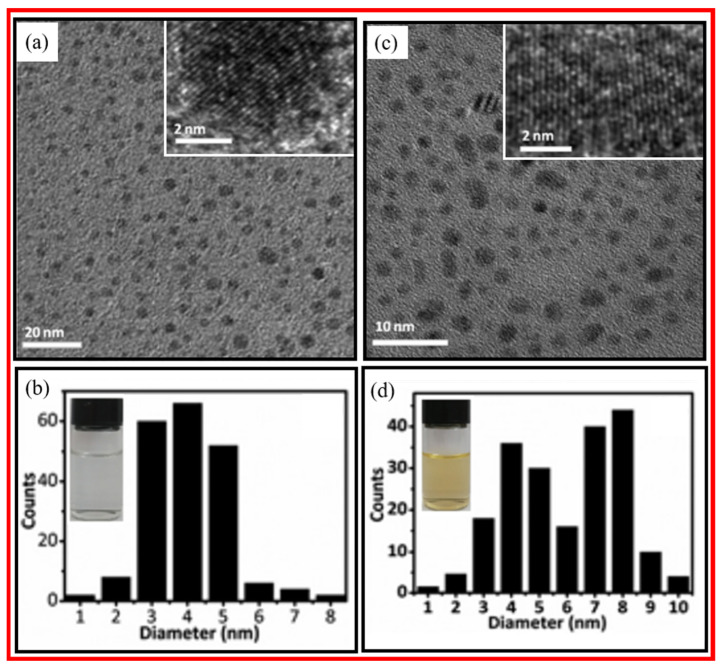
(**a**) TEM and HRTEM images and (**b**) size distribution and photograph of colorless CDs. (**c**) HRTEM and TEM images and (**d**) size distribution and photograph of bright-yellow CDs. Reproduced with permission [34].

Liu et al. prepared monodispersed and highly crystalline CDs using electrochemical oxidation of a graphite electrode precursor in an alkaline solution [34]. The CDs exhibited temperature-dependent color change such that colorless CDs synthesized at 4 °C tinted bright yellow color at room temperature. This color change was attributed to the oxygenation of CD surfaces. Figure 5a,b displays the TEM and HRTEM images, size distribution, and photograph of the colorless CDs, and Figure 5c,d exhibits the TEM and HRTEM images, size distribution, and photograph of the bright-yellow CDs. TEM images revealed that colorless CDs were monodispersed in size with an average diameter of 4.0 ± 0.2 nm, while bright-yellow CDs afforded two size distributions: one from monodispersed CDs, similar to the colorless CDs, and the other from aggregated CDs with an average diameter of 8.0 ± 0.3 nm.

#### 2.1.3. Laser Ablation

In laser ablation, a graphite precursor is exposed to laser irradiation, and CDs are produced from the precursor [35,36]. Laser ablation is classified into two categories: laser ablation in solution and that of powdered sample [36]. Notably, the double beam laser ablation method provided smaller CDs with a narrower size distribution than the single beam laser ablation method [37].

**Figure 6:**
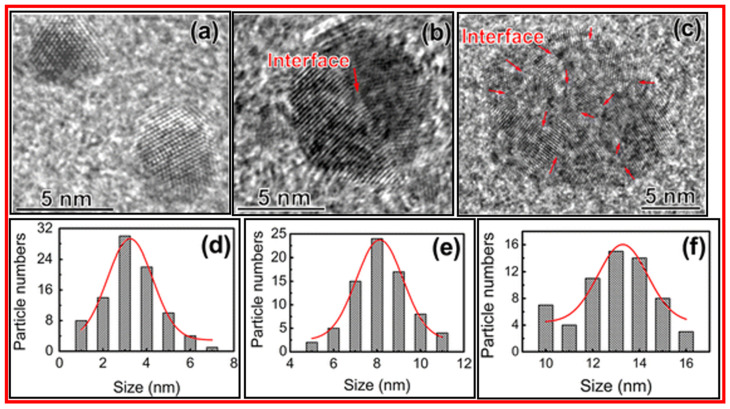
HRTEM images of CDs prepared using (**a**) 0.3, (**b**) 0.9, and (**c**) 1.5 ms laser pulse widths, respectively. (**d**–**f**) The corresponding size distributions. Reproduced with permission [38]. “Interface” (as labeled with arrows) indicates boundaries between CDs.

Hu et al. prepared CDs by laser ablation of graphite flakes in polyethylene glycol (Mn = 1500 amu) solution [38]. The size of CDs was tuned by controlling the laser pulse width. The longer laser pulse width provided a larger particle size of CDs. Figure 6a–c exhibits the HRTEM images, and Figure 6d–f displays the size distributions of CDs prepared by 0.3, 0.9, and 1.5 ms laser pulse widths, respectively. Figure 6a exhibits the single crystalline CDs, whereas CDs in Figure 6b,c are composed of two or more crystalline grains.

#### 2.1.4. Ultrasonic-Assisted Method

This method utilizes ultrasound irradiation to synthesize CDs [39]. The ultrasonic-assisted method has the advantage of low cost and simplicity in operation to synthesize CDs.

**Figure 7:**
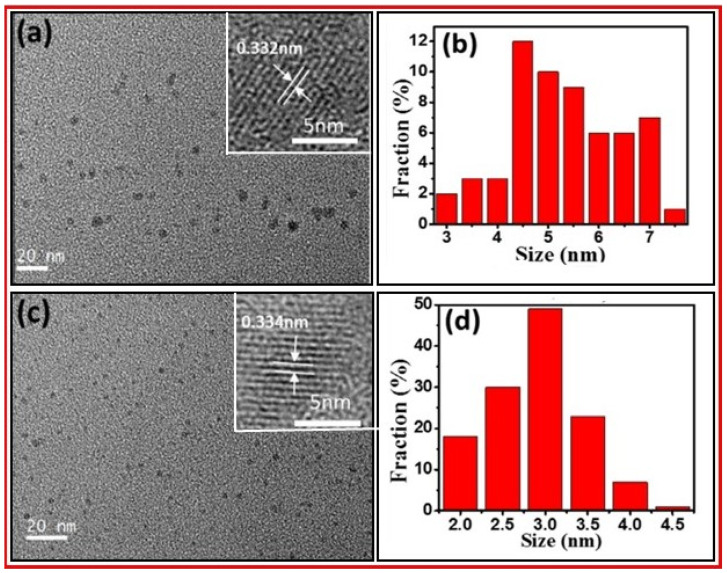
(**a**) TEM and HRTEM images and (**b**) size distribution of CDs. (**c**) TEM and HRTEM images and (**d**) size distribution of N-CDs. Reproduced with permission [40].

Wu et al. prepared water-soluble photoluminescent CDs by an ultrasonic-assisted chemical oxidation method of petroleum coke [40]. The CD surfaces were rich in oxygen-containing functional groups. Then, CDs were further treated hydrothermally in ammonia to prepare N-CDs. Figure 7a,b exhibits the TEM and HRTEM images and size distribution of CDs with an average size of 5.0 nm. Figure 7c,d displays the TEM and HRTEM images and size distribution of N-CDs with an average size of 2.7 nm. The HRTEM images of CDs and N-CDs showed their lattice spaces of 0.332 and 0.334 nm, respectively (both are in good agreement with the 002 planes of graphite).

### 2.2. Bottom-Up Approach

**Figure 8:**
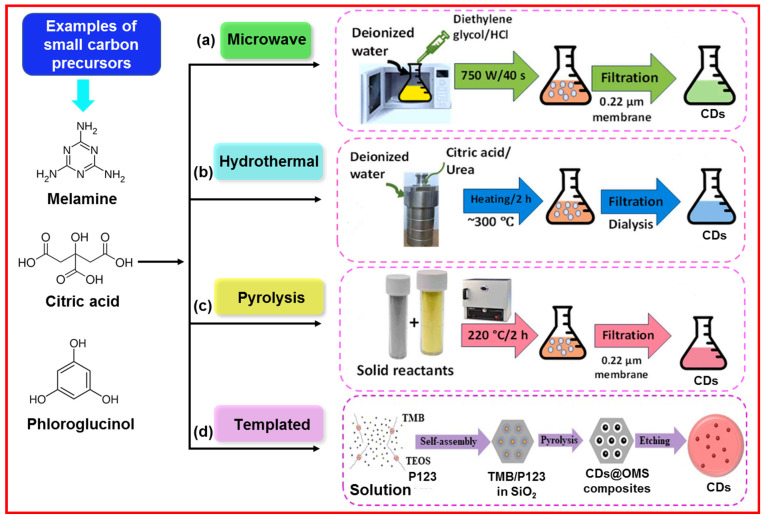
Schematic of “bottom-up” approaches to prepare CDs: (**a**) microwave [29], (**b**) hydrothermal [29], (**c**) pyrolysis [29], and (**d**) templated methods: TMB = 1,3,5-trimethylbenzene; TEOS = tetraethoxysilane; P123 = copolymer Pluronic P123; OMS = ordered mesoporous silica [43].

The bottom-up approach involves the carbonization of organic molecules as carbon sources or precursors. The carbonizing molecules are coupled together to form CDs [41]. Owing to commercial availability and facile carbonization, organic molecules with hydroxy (-OH), carboxylic acid (-COOH), and amine (-NH_2_) functional groups are generally used as precursors [42]. Figure 8 exhibits the examples of organic carbon precursors such as melamine, citric acid, and phloroglucinol as well as the synthesis of CDs through “bottom-up” approaches.

#### 2.2.1. Microwave-Assisted Method

The microwave method involves microwave irradiation of precursors to produce CDs. This method is conveniently applied to various kinds of precursors to prepare CDs in a short reaction time [44,45]. For example, Yu et al. prepared CDs using phthalic acid and triethylenediamine as precursors in a 60 s reaction time [46].

**Figure 9:**
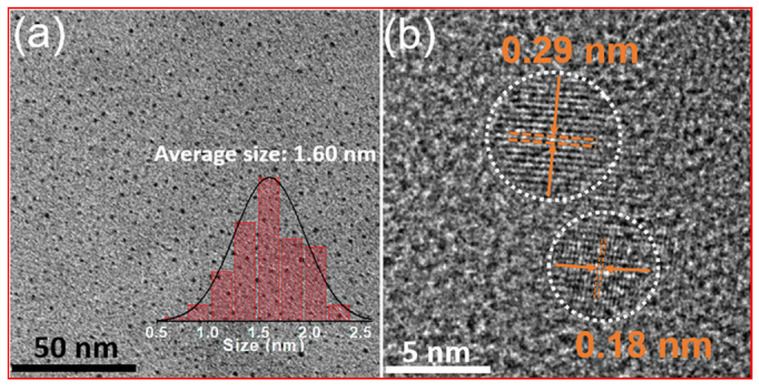
(**a**) TEM image and size distribution, and (**b**) HRTEM image and lattice fringes of CDs. Reproduced with permission [47].

Jiang et al. prepared RNA-targeting CDs by the microwave thermal decomposition method of neutral red and levofloxacin as precursors for the liver cell imaging [47]. Figure 9a,b exhibits a TEM image with size distribution and an HRTEM image of CDs, respectively. The TEM image revealed that CDs were well-dispersed with an average diameter of 1.60 nm. The HRTEM image exhibited 0.18 and 0.29 nm lattice fringes, corresponding to the (020) and (100) planes of graphitic carbon, respectively.

#### 2.2.2. Hydrothermal Method

The hydrothermal method is considered an ecofriendly, nontoxic, and cost-effective method for preparing CDs using diverse carbon sources [48,49,50,51].

**Figure 10:**
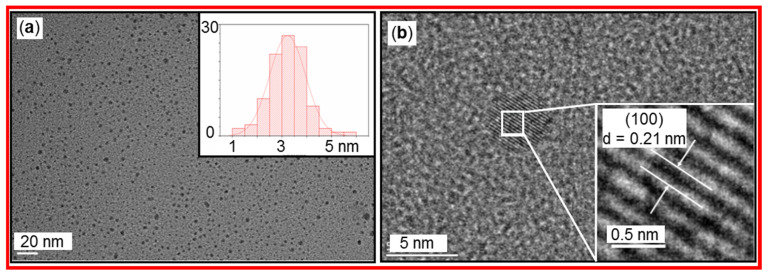
(**a**) TEM image and particle size distribution of CDs. (**b**) HRTEM image and lattice fringes of CDs. Reproduced with permission [52].

Bao et al. prepared a dual-function fluorescent CD probe by hydrothermal method using citric acid as a carbon source and *o*-phenylenediamine as a nitrogen source [52]. Figure 10a displays the TEM image and particle size distribution of the CDs with spherical morphology and good dispersion. The particle size of CDs ranged from 1.24 to 6 nm, with the average diameter of 3.23 nm. The HRTEM image exhibited that the CDs had a lattice spacing of 0.21 nm, corresponding to (100) crystal planes in the graphite phase, as depicted in Figure 10b. The CDs worked as a dual-function fluorescent probe for the detection of Fe^3+^ in the brown sugar and sunset yellow dye in beverages.

#### 2.2.3. Pyrolysis Method

This method involves thermal decomposition of carbon precursors at high temperatures to produce CDs. The pyrolysis method is considered an easy operation, low cost, solvent-free, and a fast reaction method.

**Figure 11:**
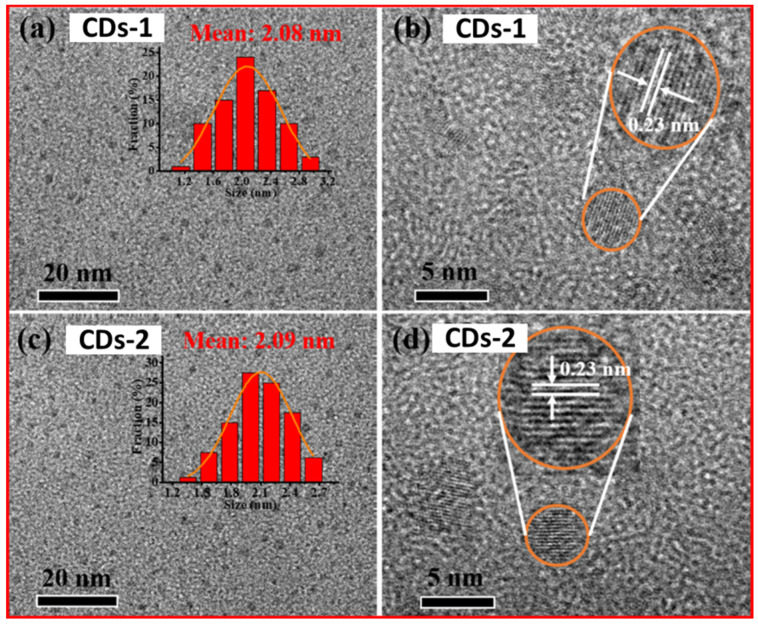
CDs-1: (**a**) TEM image with size distribution histogram (inset) and (**b**) HRTEM image with lattice fringes. CDs-2: (**c**) TEM image with size distribution histogram (inset) and (**d**) HRTEM image with lattice fringes. Reproduced with permission [53].

Wang et al. successfully prepared CDs using one-pot solid-phase pyrolysis in an autoclave (closed environment) and a crucible (open environment) [53]. The CDs prepared in an open environment had a longer emission wavelength, a higher crystallinity, and less surface state emission than CDs prepared in a closed environment. Figure 11a,b exhibits the TEM image with size distribution and the HRTEM image with lattice fringes of CDs-1, respectively, and Figure 11c,d displays the TEM with size distribution and the HRTEM image with lattice fringes of CDs-2. Both CDs were well dispersed with average sizes of 2.08 and 2.09 nm, respectively, a lattice spacing of 0.23 nm, corresponding to (100) crystal planes in the graphite phase.

#### 2.2.4. Templated Method

The templated method involves a support material (i.e., a template) to produce CDs. The size, morphology, and surface properties of CDs can be controlled using templates.

**Figure 12:**
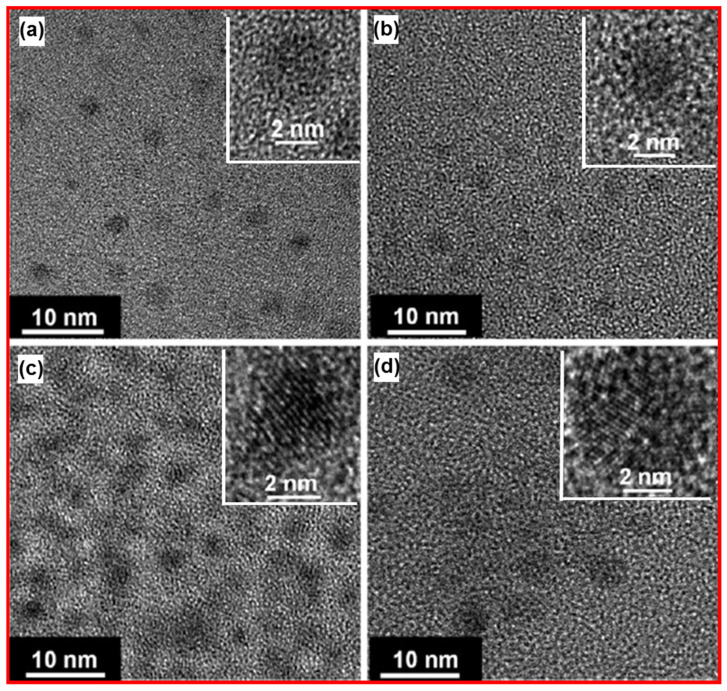
HRTEM images of CDs: (**a**) CD_TMB_, (**b**) CD_DAB_, (**c**) CD_PY_, and (**d**) CD_PHA_. Reproduced with permission [43].

Yang et al. prepared monodispersed photoluminescent CDs by the soft-hard template approach [43]. Photoluminescent CDs were prepared using copolymer Pluronic P123 as a soft template, ordered mesoporous silica (OMS) as a hard template, and 1,3,5-trimethylbenzene (TMB), diaminebenzene (DAB), pyrene (PY), and phenanthroline (PHA) as carbon sources. Figure 12a–d exhibits HRTEM images of CDs, which were prepared using TMB, DAB, PY, and PHA, respectively. The copolymers served as micelles (i.e., soft templates) to encapsulate carbon precursors, and the OMS served as a separator (i.e., hard templates) of the micelles. The HRTEM image revealed that the soft-hard templated approach prevented the aggregation of CDs during pyrolysis.

## 3. Characterizations

**Figure 13:**
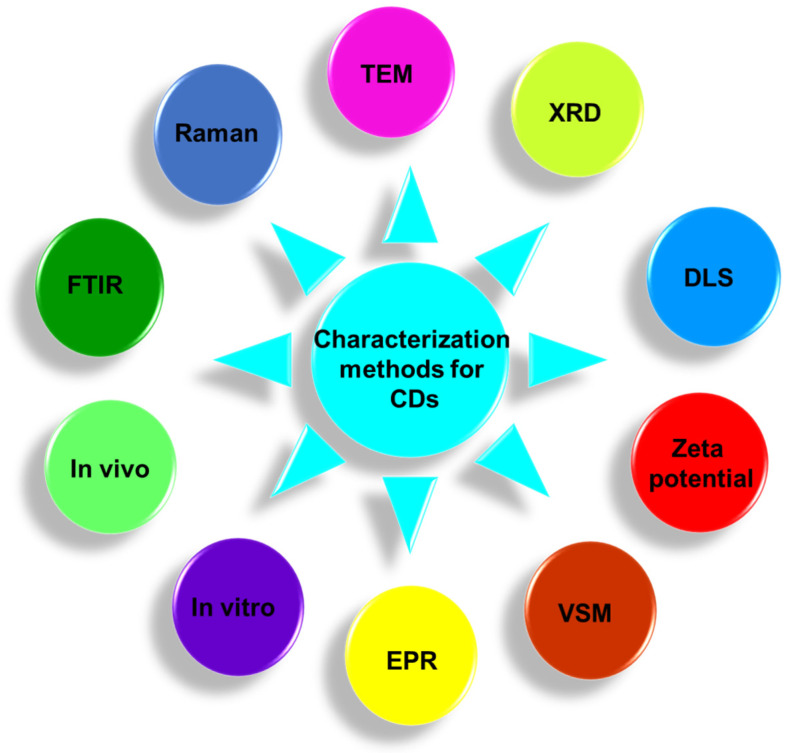
Schematic illustration of various characterization methods to elucidate various physicochemical properties of CDs.

Several analytical techniques have been used to characterize various physicochemical properties of CDs, such as the size, crystal structure, elemental composition, surface charge, hydrodynamic diameter, cytotoxicity, and magnetic properties. The characterization helps to modify and optimize the synthesis. This section will provide overviews of various characterization methods to analyze various physicochemical properties of CDs. TEM, Fourier transform infrared (FT-IR) absorption spectroscopy, Raman spectroscopy, X-ray diffraction (XRD), dynamic light scattering (DLS), zeta potential measurement, vibrating sample magnetometry (VSM), electron paramagnetic resonance (EPR) spectroscopy, in vitro, and in vivo cytotoxicity measurement are such methods to elucidate properties of CDs (Figure 13).

### 3.1. FT-IR Absorption, Raman, XRD, DLS, and Zeta Potential Analyses

**Figure 14:**
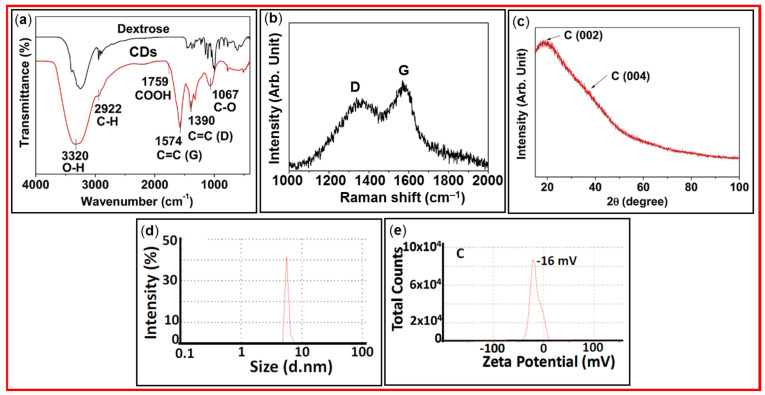
Various data of CDs: (**a**) FT-IR absorption spectra of CDs and dextrose precursor [55]. (**b**) Raman spectra [60]. (**c**) XRD pattern [55]. (**d**) DLS pattern [67]. (**e**) Zeta potential curve [67].

TEM is the primary analysis to elucidate the particle size and morphology of synthesized CDs, as illustrated in Figure 4, Figure 5, Figure 6, Figure 7, Figure 9, Figure 10, Figure 11 and Figure 12 in the synthesis parts. FT-IR absorption spectroscopy is used to identify the functional groups present on the surfaces of CDs and allows following the post-surface modification progress [54,55,56,57,58,59,60,61,62,63]. FT-IR absorption spectroscopy is suitable to measure gaseous, liquid, and solid-state samples. Figure 14a exhibits FT-IR absorption spectra of amorphous CDs and dextrose precursor [55], and Table 1 lists the FT-IR absorption wavenumbers of characteristic vibrations of CDs prepared from different carbon sources. Raman spectroscopy can be used to identify CDs and evaluate the crystalline or amorphous nature of CDs [56]. The Raman spectrum of CDs displays two distinct peaks, D- and G-bands at ~1360 and ~1580 cm^−1^, respectively, arising from sp^2^ carbons (i.e., C=C double bonds). The D-band appears due to the vibrations of defect sp^2^ graphitic carbons, whereas the G-band is the primary mode in graphene and graphite, which is due to planar sp^2^ graphitic carbons; therefore, highly crystalline graphene and graphite have a strong G- and weak D-band. Figure 14b shows the Raman spectrum of the CDs prepared from citric acid and neutral red [60], and Table 1 lists the D- and G-bands of CDs prepared from different carbon sources. XRD is used to determine the crystal structure of CDs. It also allows us to examine the chemical composition, phase purity, and particle size of CDs [64,65]. In XRD patterns, broad peaks indicate the poor degree of crystallinity (or amorphousness) of CDs. Figure 14c shows two broad peaks at 2θ = ~19° and ~38° from the graphitic carbon C(002) and C(004) crystal planes in the amorphous CDs [55]. Dynamic light scattering (DLS) allows us to examine the hydrodynamic particle diameter distribution or aggregation of CDs in aqueous media [66]. Figure 14d exhibits a DLS pattern of CDs (prepared using citric acid) with an average hydrodynamic diameter below 10 nm [67]. Zeta potential of CDs reflects their surface charge [66,68]. It helps to predict the functional groups, hydrophilicity, and electrostatic stability of the CDs in aqueous solution. For instance, CDs with -COOH groups will have negative zeta potentials, while CDs with NH_2_ groups will have positive zeta potentials [69]. Figure 14e shows the zeta potential of CDs prepared from citric acid with a value of −16 mV [67], indicating that the CDs have negatively charged surfaces owing to -COOH groups.

### 3.2. Magnetic Properties

The magnetic ordering of CDs makes them magnetic materials [70]. The magnetic ordering of CDs is due to the presence of intrinsic disorder and surface defects, providing unpaired electrons [71]. The unpaired electrons cause magnetic ordering in CDs [72].

#### 3.2.1. VSM

**Figure 15:**
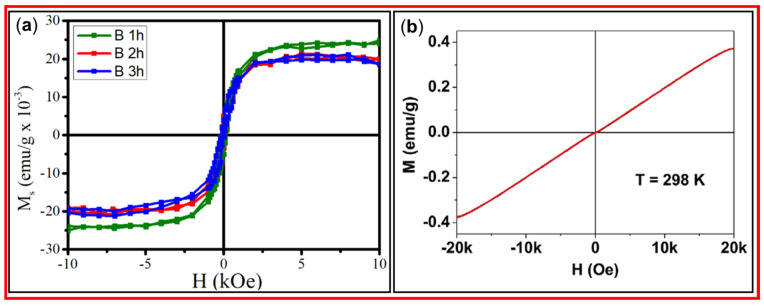
(**a**) Magnetization curves of B 1 h, B 2 h, and B 3 h CDs at room temperature (pennisteum glaucum as the precursor) [73]. (**b**) Magnetization curve of amorphous CDs at room temperature (dextrose as the carbon precursor) [55].

Tripti et al. prepared CDs using the biomass precursor pennisteum glaucum and a simple pyrolysis method [73]. Magnetic properties of the synthesized CDs, i.e., B 1 h, B 2 h, and B 3 h, where “B” represents the biomass precursor and “1 h”, “2 h”, and “3 h” represent the pyrolysis times, were investigated. The magnetic ordering was attributed to the interaction of unpaired electrons. Figure 15a shows the saturation magnetization values of 0.02412, 0.02006, and 0.01872 emu/g for B 1 h, B 2 h, and B 3 h CDs, respectively, revealing that as the pyrolysis time increased, the saturation magnetization decreased; this implies that defect structures and unpaired electrons increased with increasing pyrolysis time. Tegafaw et al. prepared amorphous CDs with an average diameter of 2.2 nm using a dextrose precursor and a wet-chemical method [55]. The M–H curve of the CDs revealed that the amorphous CDs had a weak paramagnetic property at room temperature, as depicted in Figure 15b [55]. Therefore, the magnetic properties of CDs depend on the synthesis method and carbon precursor.

#### 3.2.2. EPR

**Figure 16:**
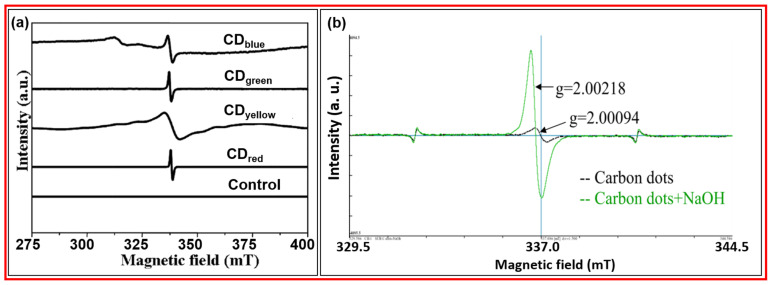
(**a**) EPR spectra of four different CDs at 25 °C; control corresponds to CDs with poor fluorescence [74]. (**b**) EPR spectra of CDs before (black) and after (green) NaOH addition to solution [75].

Bhunia et al. prepared fluorescent CDs using carbohydrate derivatives and chemical methods [74]. Four kinds of fluorescent CDs with different emission colors were prepared by changing synthesis conditions; they were CD_blue_, CD_green_, CD_yellow_, and CD_red_. Figure 16a displays the EPR spectra of four kinds of CDs with different emission colors at 25 °C, confirming the existence of free electrons in CDs. Zhao et al. prepared CDs with a 2–4 nm diameter using a microwave approach and glucose and PEG 1500 as the carbon sources [75]. Figure 16b exhibits the EPR spectra of CDs. g-value of 2.00094 revealed that the ground state of CDs was singly occupied in the orbital. When NaOH was added into the CD solution, the peak intensity and area increased, indicating more singly occupied orbitals by free electrons in CDs.

### 3.3. In Vitro and In Vivo Cytotoxicity

CDs are potential candidates in biological and biomedical applications owing to their very low or nontoxic performance [76,77,78]. Furthermore, their cytotoxicity can be improved through surface modifications [79].

**Figure 17:**
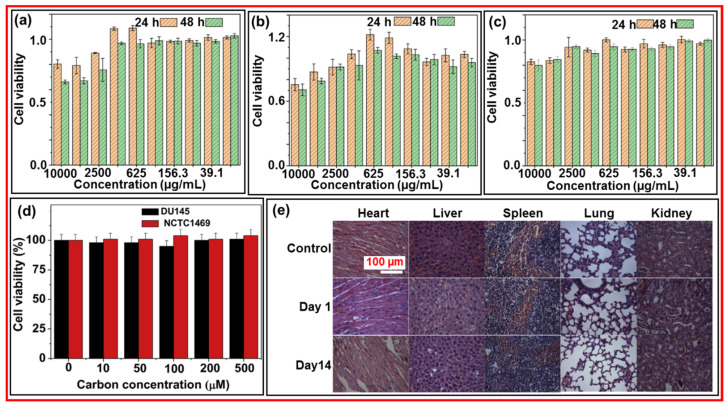
In vitro cytotoxicity of CDs: (**a**) RAW 264.7, (**b**) L02, (**c**) 293T [80], and (**d**) DU145 and NCTC1469 cells [55]. (**e**) Hematoxylin and eosin-stained tissue slices (liver, spleen, kidney, heart, and lung) of mice at 1 and 14 days after injection (dose = 23 mg CDs/kg) [81].

Wang et al. prepared ultrasmall and highly biocompatible CDs using the natural plant *Pollen Typhae* (PT) and a one-pot pyrolysis method [80]. Figure 17a–c exhibits the cell viability of mouse macrophage tumor (RAW 264.7), cervical cancer line HeLa derivative (L02), and human embryonic kidney (293T) cells, respectively, at the concentration range from 19.53 to 2500 µg CDs/mL, displaying almost no cellular toxicity. Figure 17d presents the in vitro cytotoxicity of amorphous CDs using human prostate cancer (DU145) and normal mouse hepatocyte (NCTC1469) cells, indicating nontoxicity up to the treated carbon concentration of 500 µM in both cells [55]. Wang et al. explored the in vivo toxicity of CDs in various organs (i.e., heart, liver, spleen, lung, and kidneys) 1 and 14 days after injection of CD solution into rats [81]. Figure 17e shows no significant histological changes in the organs after injection as compared with those of the control, confirming the nontoxicity of the CDs.

**Figure 18:**
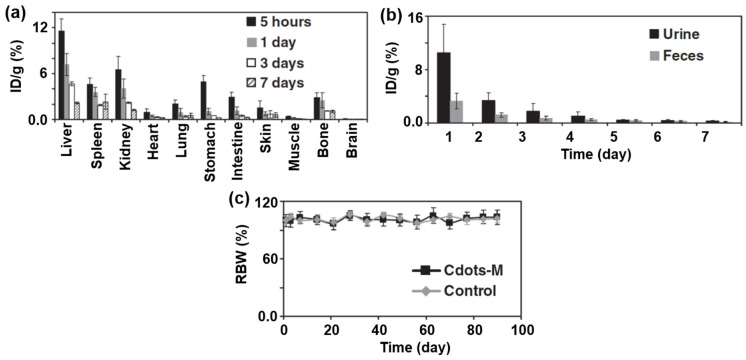
(**a**) Biodistribution of CDs at different times after sacrificing mice (IV injection dose = 4 mg/kg). ID/g (%) indicates the percentage of injected dose per gram of tissue. (**b**) Distribution of CDs in urine and feces over time. (**c**) Relative body weight (RBW) (%) of mice after IV injection (20 mg/kg) up to 90 days [30].

Tao et al. measured the time-dependent biodistribution of CDs by sacrificing mice at 5 h, 1 day, 3 days, and 7 days after IV injection (injection dose = 4 mg/kg) (Figure 18a) [30]. They found that the CDs were mainly accumulated in the reticuloendothelial system, such as the liver and spleen. The initial uptake of CDs at the kidneys was high, indicating that the CDs could pass the glomerulus and be excreted by urine, owing to their ultrasmall particle size (2–5 nm). The obvious trend of decreasing CD amounts with time in biodistribution indicated that the CDs were cleared out from the mice’s body. Furthermore, the time-dependent distribution of CDs in urine and feces indicated that the clearance of CDs was possibly through both renal and fecal excretions (Figure 18b). For long-term in vivo toxicity, they investigated the relative body weight (RBW) (%) of mice over 90 days and did not notice any body weight drop for the treatment group (IV injection dose = 20 mg/kg), similar to the untreated control group (Figure 18c). In addition, they did not see any toxic effects of CDs in hematology analysis up to 90 days, demonstrating the long-term nontoxicity of CDs.

For clearance of CDs, the renal (as urine) and hepatic (bile to feces) pathways are the two major excretion routes [82]. The ultrasmall CDs (<5 nm) can be excreted through the renal system, whereas large CDs (>5 nm) can be excreted through the hepatic pathway. For example, as shown in higher intensities in urine in Figure 18b, the CDs (2–5 nm) were mainly excreted through the renal system owing to their ultrasmall particle size [30]. The renal excretion takes hours to days after IV injection, whereas the hepatic excretion takes hours to months. If CDs do not degrade and cannot be excreted through the renal and hepatic routes, they may be processed by the mononuclear phagocyte system with retention in the body for months to years, with the possibility of generating toxicity.

## 4. CDs as diaCEST MRI Contrast Agents

### 4.1. Principle of CEST

The advancement of MRI was transformed with the development of contrast agents because they improved the images and enhanced diagnostic precision through contrast enhancements. Until now, various kinds of metal-based MRI contrast agents have been developed; these are Gd(III)-chelates, Mn(II)-chelates, and iron oxide nanoparticles [83,84,85,86,87,88,89]. However, these metal-based MRI contrast agents are restricted to low-concentration injections owing to their toxicity. Therefore, metal-free MRI contrast agents such as CEST MRI contrast agents have been recently introduced [90,91,92,93,94,95].

**Figure 19:**
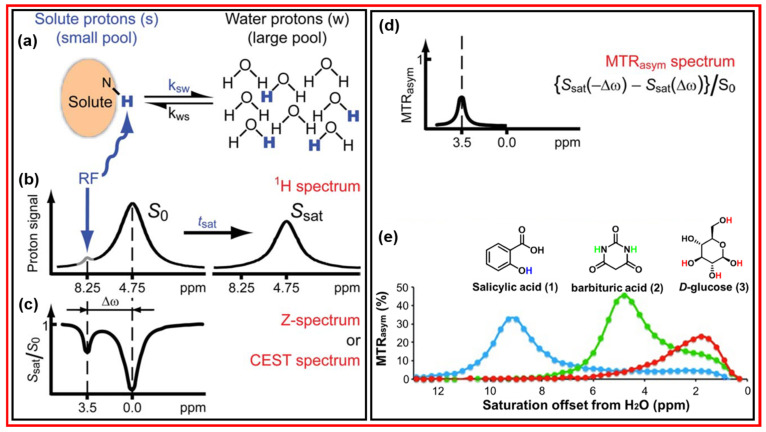
(**a**) Principle of the CEST mechanism: the saturated solute protons are exchanged with bulk water protons at the rate *K*_sw,_ and the unsaturated bulk water protons return to the solutes at the rate *K*_ws_. Measurement of CEST: (**b**) solute protons are saturated at their specific resonance frequency at 8.25 ppm and bulk water protons at 4.75 ppm with the signal intensity S_0_ (left spectrum), and the proton exchange leads to the bulk water proton signal reduction with a constant signal intensity S_sat_ after a period (t_sat_) (right spectrum); (**c**) normalized proton signal spectrum, called the Z-spectrum or CEST spectrum (Δω is the resonance frequency shift in solute protons with respect to the water proton resonance frequency); and (**d**) MTR asymmetry (MTR_asym_) plot of the Z-spectrum after removing the effect of the bulk water proton signal [91]. (**e**) MTR_asym_ plots in % for the three agents: salicylic acid (1), barbituric acid (2), and D-glucose (3) [96].

Figure 19a displays the principle of the CEST mechanism in which the saturated solute protons are exchanged with bulk water protons at the rate *K*_sw_, and the unsaturated bulk water protons return to the solutes at the rate *K*_ws_ [91,95]. The left spectrum in Figure 19b presents the solute protons, which resonate at a different frequency from that of bulk water protons with a signal intensity of S_0_. The saturated solute protons at a specific resonance frequency are transferred to bulk water through exchange with unsaturated water protons, decreasing the water proton resonance signal to a constant signal intensity S_sat_ after a period (t_sat_), as depicted in the right spectrum in Figure 19b. Figure 19c exhibits the normalized proton spectrum; this spectrum is called the Z-spectrum or CEST spectrum; the resonance frequency shift in solute protons is written as Δω with respect to the water proton resonance frequency. Figure 19d displays the result of magnetization transfer ratio (MTR) asymmetry analysis in % of the Z-spectrum after removing the effect of water proton signals using the formula {S_sat_(−Δω) − S_sat_ (Δω)}/S_0_. Figure 19e displays the chemical shift in various exchangeable proton sources and their MTR efficiencies [96], showing that the higher MTR efficiency and the higher saturation offset from H_2_O will provide sharper and stronger CEST signals. Therefore, the effective CEST MRI contrast agents should have high MTR efficiency and a high saturation offset from H_2_O.

### 4.2. Applications of CDs as diaCEST MRI Contrast Agents

The CEST MRI contrast agents can be divided into two categories based on their composition: paramagnetic CEST (paraCEST) agents [26,97,98] and diamagnetic CEST (diaCEST) agents [19,20,21,22]. The metal-free CDs can be used as diaCEST MRI contrast agents to amplify MRI contrast efficiency.

**Figure 20:**
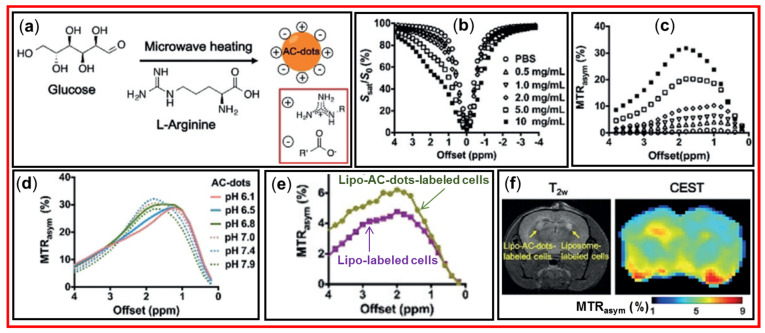
(**a**) Synthesis of AC-dots. (**b**) Ac-dot concentration (mg/mL) dependent Z-spectra. (**c**) Ac-dot concentration-dependent MTR_asym_ plots at pH = 7.4. (**d**) pH-dependent MTR_asym_ plots of AC-dots (10 mg/mL) in PBS. (**e**) MTR_asym_ plots of Lipo-AC-dots-labeled cells and Lipo-labeled cells as control. (**f**) T_2_-weighted (T_2w_) MR image (left) and corresponding CEST image (right) at 2 ppm of a mouse brain at 24 h after implantation [19].

Zhang et al. prepared arginine-modified carbon dots (AC-dots) as a new class of diaCEST MRI contrast agents [19]. Figure 20a shows the synthesis of AC-dots with an average diameter of 4.7 nm using glucose and arginine as precursors and microwave irradiation. The arginine was used to modify the surfaces of CDs. Figure 20b,c displays the Z-spectra and MTR_asym_ plots, respectively, with an increment of AC-dot concentration in which MTR_asym_ plots exhibited the signal increment with the increase in AC-dot concentration. Figure 20d exhibits that MTR_asym_ plots at pH = 6.1 and 6.5 had maximum signals at ~1 ppm owing to hydroxyl protons of AC-dots, but at pH ≥ 7, maximum signals were observed at ~2 ppm because the CEST signals were replaced into guanidinium protons. Figure 20e,f shows that liposome (Lipo)-AC-dot-labeled cells had higher CEST contrast enhancements than the control liposome-labeled cells. Importantly, as shown in Figure 20f, the T_2_-weighted (T_2w_) MR images showed similar contrasts at the left and right mouse brains, but the CEST image at 2 ppm (MTR_asym_ = ~6%) showed higher contrasts at the left brain (Lipo-AC-dot-labeled cells injected) than the right brain (control Lipo-labeled cells injected). This work clearly demonstrated the effectiveness of the CDs as a new class of diaCEST MRI contrast agents in sensitively detecting diseases with minimal artificial defect contrasts.

**Figure 21:**
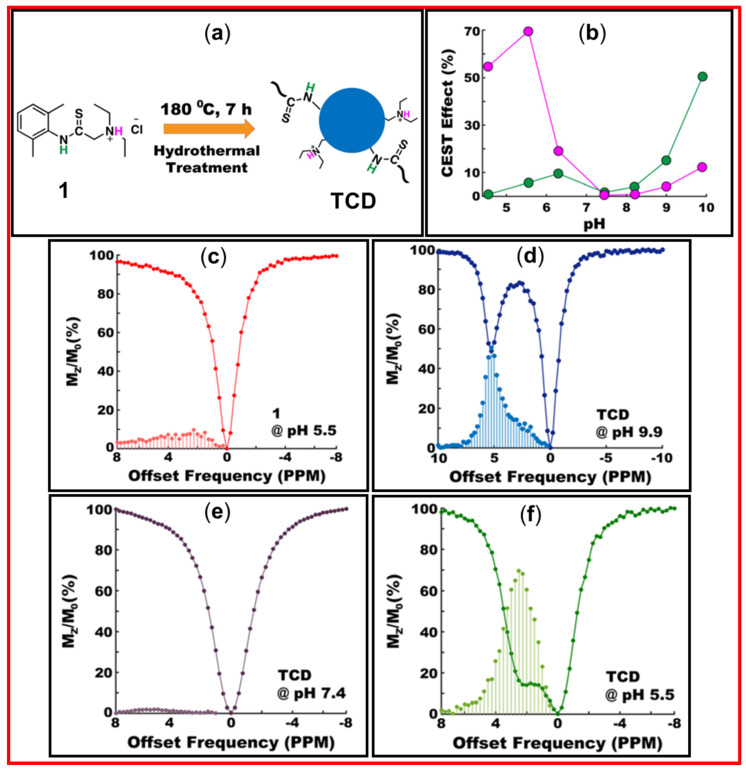
(**a**) Synthesis of TCDs from the carbon precursor “**1**” showing amide (green) and ammonium (pink) protons exchangeable with bulk water protons in diaCEST MRI, (**b**) CEST effects (%), i.e., maximum MTR_asym_ of the precursor estimated from Z-spectra for the two types of protons at different pH values. (**c**) Z-spectrum (i.e., M_z_/M_0_) and MTR_asym_ of the precursor at pH = 5.5. Z-spectra and MTR_asym_ of the TCDs at pH = (**d**) 9.9, (**e**) 7.4, and (**f**) 5.5 [20].

Pandey et al. prepared water-soluble thioamide-based CDs (TCDs) as diaCEST MRI contrast agents [20]. The amino-thioamide precursor (“**1**”) was ineffective as a diaCEST MRI contrast agent owing to its poor water solubility. However, TCDs prepared using thermal treatment served as diaCEST contrast agents owing to their improved water solubility. Figure 21a exhibits the synthesis of TCDs using hydrothermal treatment, and Figure 21b displays the CEST effect (i.e., maximum MTR_asym_) as a function of pH for the amide (green) and ammonium (pink) exchangeable protons in Figure 21a. As shown in Figure 21c, the precursor “**1**” in PBS at pH = 5.5 exhibited a broad diaCEST or Z-spectrum in terms of M_z_/M_0_ (%) (M_0_ and M_z_ are the water proton signal intensities before and after saturation), corresponding to S_sat_/S_0_ in Figure 19c, with 9.7% maximum efficiency of MTR_asym_ at Δω = 2.25 ppm. The CEST results of TCDs are presented in Figure 21d–f. At pH = 9.9, a strong and sharp diaCEST signal with 50.3% MTR_asym_ was observed from amide protons of TCDs at Δω = 5.25 ppm (Figure 21d). However, as shown in Figure 21e, the diaCEST efficiency was poor at physiological pH = 7.4. However, improved diaCEST efficiency was obtained at physiological pH by the variation in the reaction time, temperature, and precursor concentration [21]. At pH = 5.5, the TCDs exhibited the maximum MTR_asym_ of ~69% from ammonium protons owing to improved water solubility of CDs (Figure 21f). This pH-dependent CEST experiment indicated that the best CEST image using TCDs prepared using an amino-thioamide precursor could be obtained at pH = 9.9.

The CDs negligibly affect longitudinal (T_1_) and transverse (T_2_) water proton spin relaxation times [19,55] because they are diamagnetic. The CDs should possess high proton exchange rates (k_sw_) with bulk water protons. In addition, the proton resonance frequency of CDs should be far from the water proton resonance frequency (i.e., the resonance frequency difference, Δω, should be high) because the k_sw_ is limited by the condition Δω ≥ k_sw_ to effectively observe the CEST effect [97]. Therefore, the effectiveness of CDs as diaCEST MRI contrast agents can be improved by maximizing both Δω and k_sw_. The in vivo imaging performance of the diaCEST MRI contrast agents can be expressed using the CEST efficiency (i.e., MTR_asym_); to obtain high MTR_asym_, Δω and k_sw_ should be high as mentioned above. In addition, the CDs should have a large amount of exchangeable protons on their surfaces, which can be achieved using proper carbon precursors with a large amount of -OH or -COOH or -NH_2_ groups in synthesis.

### 4.3. Comparison with Other MRI Contrast Agents

The conventional and CEST MRI contrast agents are classified in Table 2 for comparison. As provided in Table 2, the operational principle of CEST MRI contrast agents is different from that of conventional MRI contrast agents. The conventional MRI contrast agents accelerate water proton spin relaxations at their accumulated regions, providing positive (T_1_) or negative (T_2_) contrasts in MR images; their imaging performance relies on longitudinal (r_1_) and transverse (r_2_) water proton spin relaxivities and r_2_/r_1_ ratios. However, the working principle of CEST MRI contrast agents is based on proton exchange with bulk water protons as described in Section 4.1; the CEST MRI contrast agents with higher proton exchange rates (k_sw_) and larger Δω (Δω ≥ k_sw_) will provide higher MTR_asym_ values and thus, higher contrasts in MR images at their accumulated regions. The conventional and paraCEST MRI contrast agents contain metal ions, whereas the diaCEST MRI contrast agents contain only nonmetals. Therefore, the diaCEST MRI contrast agents are generally less toxic, more biocompatible, and safer for medical applications than metal-based MRI contrast agents. However, the sensitivity of CEST MRI contrast agents is lower than that of conventional MRI contrast agents [19,99], requiring higher IV injection doses compared with Gd(III)-chelates [100]. Therefore, their sensitivity improvement will make them the next generation of MRI contrast agents as an alternative to Gd(III)-chelates for clinical applications.

## 5. Conclusions and Future Perspectives

This review overviewed the progress and advancements of the synthesis, characterizations, and MRI application of CDs as diaCEST MRI contrast agents. As reviewed here, only a few studies on diaCEST MRI contrast agents based on CDs exist. Nonetheless, the CDs demonstrated the excellent performance suitable for applications as a new class of nontoxic and next-generation MRI, i.e., diaCEST MRI contrast agents.

The CDs have received great attention owing to their great potential for biomedical applications [101,102,103]. For practical application of CDs in MRI, the CDs should be highly reproducible in synthesis to obtain constant magnetic properties and stable CEST signals over time. In addition, mass production of CDs is necessary for their practical applications in MRI. Until now, CDs have been synthesized using various methods with explanations of their plausible formation mechanisms. Although numerous synthetic methods have been introduced, a standard synthetic methodology producing high-quality CDs with the required morphology, size, properties, and surface functional groups has not been developed. Therefore, future research should address this issue to improve and optimize the performance and applications of CDs. Above all, the synthesis should satisfy the high water solubility of CDs with many exchangeable protons with bulk water protons to apply them as effective diaCEST MRI contrast agents.

To improve the MR image quality, contrast agents can be used [104]. As reviewed here, the diaCEST MRI contrast agents correspond to a new class of MRI contrast agents. They do not rely on metal ions but on exchangeable protons with bulk water protons. The diaCEST MRI technique can provide resonance frequency selectivity because the resonance comes from exchangeable protons of materials, but not the bulk water protons, providing image contrasts with minimal artificial defects from bulk water proton signals. In addition, compared with conventional Gd-chelates [105] and iron oxide-based superparamagnetic nanoparticles [106], diaCEST MRI contrast agents have considerably lower biotoxicity because they are made of nontoxic elements such as C, H, O, and N.

The CD-based diaCEST MRI contrast agents can provide several advantages over conventional MRI contrast agents. Besides non-toxicity and resonance frequency selectivity, they can be easily synthesized using various carbon precursors and various synthetic methods. They can be made highly hydrophilic with many exchangeable protons with bulk water protons. Furthermore, their surfaces can be easily modified to conjugate with various functional molecules such as targeting ligands and drugs to increase specificity and treat diseases. The present status of CD-based diaCEST MRI contrast agents is just beginning at the research level, as can be evidenced from only a few research papers published so far. However, based on previous reports, the future of CD-based diaCEST MRI contrast agents is very promising. The high sensitivity and frequency selectivity of the CD-based diaCEST MRI contrast agents will allow us to detect and monitor diseases at the molecular level. Therefore, metal-free CDs as promising potential diaCEST MRI contrast agents will open a new journey to MRI.

## Figures and Tables

**Table 1:** FT-IR absorption and Raman shift (cm^−1^) of CDs.

Carbon Precursor	FT-IR (cm^−1^)		Raman Shift (cm^−1^)		Ref.
	Wavenumber	Vibration Mode	D-Band	G-Band	
Sodium citrate and polyacrylamide	3436 and 1410	N-H/O-H stretching and O-H bending, respectively	1363	1582	[57]
	1590	N-H bending or asymmetric stretching of carboxylate anions			
	1648 and 1059	C=O stretching and C-N stretching, respectively			
L-ascorbic acid and β-alanine	1720	C=O stretching	1365	1595	[58]
	1370	O-H bending			
	1214	C-O stretching			
	1050	C-N stretching			
Glucose and *m*-phenylenediamine	3400	N-H/O-H stretching	1357	1565	[59]
	1605	C=N or C=O stretching			
	1137	Benzene C-H stretching			
Mandelic acid and ethylenediamine	3352 to 3031	O-H and N-H stretching	1358	1574	[60]
	2926 and 1367	C-H stretching and bending, respectively			
	1570	C=O stretching			
	1059	C-O stretching			
	692	N-H deformation			
Oatmeal	3432	O-H/N-H stretching	1359	1584	[61]
	2921	C-H stretching			
	1625 and 1382	C=O asymmetric and symmetric stretching, respectively			
	1241 and 1151	C-N and C-OH stretching, respectively			
	1091	C-O stretching			
Lychee seeds	3443	O-H or N-H stretching	1387	1585	[62]
	2981	C-H stretching			
	1633	C=O stretching			
	1055	C-O stretching			
Citric acid and neutral red	3496	O-H stretching	1340	1596	[63]
	1720	C=O stretching			
	1210	C-O-C stretching			
	3296	N-H stretching			
	1551 and 1412	C=C and C-N stretching, respectively			

**Table 2:** Classification of MRI contrast agents.

Classification	Operational Principle	Chemical	Contrast	Imaging Performance Parameter	Sensitivity	Clinical Usage
Conventional	Acceleration of water proton spin relaxations	metal chelates such as Gd(III)-chelates, Mn(II)-chelates, Fe(III)-chelate	Positive (T_1_) or negative (T_2_)	r_1_, r_2_/r_1_	High	Gd(III)-chelates
		metal oxide nanoparticles such as Gd_2_O_3_, Fe_3_O_4_, MnO				
CEST	paraCEST (water exchange)	Eu(III)-chelates, Dy(III)-chelates, Tm(III)-chelates	MTR_asym_	k_sw_, Δω	Low	-
	diaCEST (proton exchange)	molecules and CDs with functional groups of -OH, -COOH, -NH_2_				

## Data Availability

Not applicable.
